# Collectanea

**Published:** 1828-04

**Authors:** 


### COLLECTANEA.

Floriferis ut apes in saltibus omnia libant,

Omnia nos, itidem, depascimur aurea dicta.

### PHYSIOLOGY.

*Dentition.*—At a late meeting of the Academie de Medicine, M. Reveillé Pariset read a note on the eruption of the teeth, and its anomalies. He cites, after Borelli, the case of a woman of sixty years, who never had teeth. Louis XIVth and Mirabeau were born with teeth; and very recently a girl was born with two canine teeth, the others made their appearance a few days after. It has been said that the appearance of teeth before birth is a sign of strength, vigour, and longevity. M. Oudet states that, in the course of dissections, he had sometimes met in fœtuses the rudiments of the teeth in a state of suppuration, which might account for the case of the woman mentioned by Borelli as having never had teeth. M. Moreau stated that the being born with teeth in the mouth was far from being really a sign of strength or longevity; for he had seen a fœtus of seven months come into the world with seven teeth, and died some little time after. *(Revue Med.)*

### PATHOLOGY.

*A Case of the Foramen Ovale remaining open in the Adult, with a general Narrowing of the Aorta and Arteries, without the Morbus Cyanosus.*—It has generally been supposed that the cause of the blue appearance of the skin in the morbus cyanosus was the aperture between the auricles of the heart continuing pervious after birth. M. Miguel has lately met with a case where this communication continued open, and yet there was no blueness of the skin; proving that the generally supposed cause of the discoloration is not correct. The case is in itself interesting.

Jean Antoine Adam, a jeweller, æt. thirty-six, came into La Charité, under M. Cayol, August 1825. This man was fair, of small stature, and had never suffered from any severe disease; his complexion was red and white. When young, he was subject to occasional difficulties of breathing, coming on in fits, once terminating in syncope: antispasmodics usually removed them. He was married, and had several children. At the age of thirty, without any apparent cause, the fits of difficulty of breathing became more intense and more frequent. During eight days, he had daily three or four attacks, each appearing likely to terminate his existence. His face suddenly reddened, acute pain in the heart and head, with palpitations, continuing for about a minute; then he grew pale, and fainted. When the attack passed, he felt quite well, till another came on. This state (strange to say!) was looked upon as the indication of some pernicious fever, and large doses of quina were administered to stop its course, but which increased it. After continuing thus during a week between life and death, a change took place: the difficulty of breathing, which had been periodical, became constant, and increased in violence; and the headache became intense. Venesection was prescribed, and the man felt himself resuscitated by the operation, ("Je ressuscitai a mesure que mon sang coulait.")

For some weeks he was relieved, when the symptoms became again aggravated. He was again bled, and with similar success; and to this frequent bleeding, and the application of leeches, he owed the preservation of his life for six years. He says himself that there were more than 6000 leeches applied, and that he was bled seventy-two times during that period.

At the present time he presents the following symptoms: Face pale and emaciated, without the least appearance of the violet or blue colour of the lips, cheeks, ears, or any part of the body; extreme anxiety; respiration short, oppressed, and laborious; orthopnœa; short, teazing cough; the chest resonant on all points, and the respiratory murmur apparent throughout; the whole body is agitated by violent palpitations of the heart; the pulse was small, unequal, intermittent; the limbs œdematous; the abdomen large and tense, and gave an evident fluctuation; the extremities were cold, and the general temperature of the body was under the natural standard; the bowels inclined to constipation.

M. Cayol, in his diagnosis, stated that there was considerable hypertrophy of the left ventricle, with narrowing of the orifice of the aorta by a commencing ossification of the sigmoid valves. A diuretic mixture, with half a drachm of acetate of potash, was prescribed, with half a drachm of Aq. Lauri Cerasi.

The patient went on getting worse and worse. The smoking Datura Stramonium was tried, and for a short time gave much relief. The powder of Belladonna was also tried, and it appeared to sooth him a little.

On the 11th of September he died, and the body was opened thirty-six hours after death. On opening the chest, the heart, distending the pericardium, and pressing upon the left lung, appeared twice the natural size. The right ventricle and auricle contained some clots of black blood: their size was larger than natural. The auricles communicated by an opening as large as a two-franc piece, evidently the foramen ovale, not obliterated. (*Revue Med.*)

---

*Hereditary Predisposition to Cataract.*—Madam St. Pierre, accompanied by part of her family, presented herself at the public consultation of M. Dupuytren. Among the individuals of this family, so many are affected with cataract, that it is difficult to explain the occurrence without referring it to hereditary disposition. At the age of upwards of sixty, Madam St. Pierre's sight first began to be affected; eighteen months afterwards, both the crystalline humors were entirely opaque. The operation of depression was performed on one eye by M. D. with perfect success. No operation was performed on the other eye.

At twenty-eight years of age, the sight of this person's daughter began to fail; soon afterwards she became unable to do more than merely distinguish day from night; the pupils were moveable, and the eye otherwise sound. Two years afterwards, M. D. depressed the cataract in one eye: the patient recovered her sight, and ten years afterwards the vision continued to be perfect. Some time afterwards, hearing of the reputation of M. Forleuze, she applied to him, and had the cataract of the other eye extracted: unfortunately, inflammation ensued, which went on to produce total opacity of the cornea, and the patient lost the eye entirely; but the inflammation produced no ill effect upon the eye formerly operated upon by M. Dupuytren.

The son of this woman, seventeen years old, was already suffering from two cataracts: they were both depressed at the Hôtel Dieu, and the lad recovered his sight.

At the time this lad was admitted into the hospital, Madame St. Pierre also brought another of her grand-children, the chrystallines of whose eyes had begun to be opaque; and one little girl besides, who complained of seeing all objects through a mist, which, as every one knows, is one of the earliest symptoms of cataract. Thus it appears that a grand-mother, her daughter, and three grand-children, were all affected with the disease.

---

### PRACTICAL MEDICINE.

*Treatment of Epilepsy.*—For several years many experiments have been tried in Germany, which appear to prove the anti-epileptic powers of the Artemesia Vulgaris. By many authors its virtues have been much extolled. Dr. BURDAL, of Wiebal, and Professor HUFELAND, have used it, gathered in October, and reduced to powder, with much success in many cases of epilepsy. The dose is from fifty to seventy grains. It has been generally given in warm small-beer. A copious perspiration mostly follows, and a few doses have frequently succeeded in effecting a cure. The cases in which the artemesia has been most beneficial, have been those in which the paroxysms of the disease occurred frequently and periodically. Very satisfactory evidence is adduced upon this subject by E. GRAEFE, who speaks confidently of the anti-epileptic powers of this medicine. According to the latter authority, it has been used with equal success in cases of catalepsy, but requires to be continued for a longer time than in epilepsy. Neither the infusion nor the decoction of the root can be depended upon. In one case of epilepsy recorded by Graefe, the patient had been afflicted with the disease for several years. He took two ounces of the powdered artemesia, in divided doses. The violence of the paroxysms gradually subsided, and in one month from the first employment of the remedy they entirely ceased. In the second case of a cataleptic woman, it was equally successful, after various other means had been employed in vain. This patient took altogether four ounces of the powder. (*Hufeland's Jour.*)

The following case is related by Dr. BONA, of Platow: A man, forty-one years of age, had been subject to epilepsy for thirty years. The cause of the original attack was unknown. The paroxysms at first occurred at long intervals of time, but at length they appeared about every fourteen days, and lasted a considerable time. Being left alone in a room with a fire, he was seized with a fit, and fell under the grate. He was severely burned in the left thigh, and extensive ulceration followed. By appropriate treatment, the parts healed, and from this time the patient remained free from attacks of epilepsy. (HUFELAND'S *Journal.*) another remedy

Dr. URBAN, of Bernstadt, recommends the Ammoniacal Sulphate of Copper in epilepsy, when purely nervous. He relates five cases in which its employment has been entirely successful. The first was that of a young man, in whom the fits were renewed every fortnight, unaccompanied with any other affection, excepting a spitting of blood, which was combated in the first place with bleeding and other antiphlogistic means. The attacks of epilepsy afterwards persisting with their former violence, Dr. Urban prescribed eight grains of the ammoniacal sulphate of copper, to be mixed with twenty-four grains of sugar and twenty-four grains of crumb of bread, and formed into forty-eight pills, of which the patient took three morning and evening, increasing the dose by one pill every two days. The young man had only five fits from this period, each at a longer interval than the preceding, and he has now remained well two years.

In the second case, the epilepsy was produced by the sudden intelligence of the woman's husband having been assassinated, and the medicine was given in rather smaller doses, and in the form of powders. The first six powders, however, produced nausea and vomiting, and it became necessary to omit them for a day. They were afterwards resumed in the same dose, which was at length increased to the amount of one grain of the salt in the day. It is to be remarked, that in this case the attacks were not diminished in frequency for some time, but they soon became of shorter duration and less violence. She finally recovered, after having taken twenty-six grains of the salt altogether. (*Ibid.*)

---

*Delirium Tremens.*—Dr. Coates has made some observations on this subject in a recent Number of the *North American Medical Journal*, from which he draws the following conclusions:

1. The disease is a delirium, and not a mania; and this distinction should be attended to, both for medical and legal reasons.

2. It consists in a heightened activity of the sensorium; and this appears to arise from the generation, in that organ, of an unusual vital power, which is not, as in common, exhausted by the narcotic poisons habitually used. This is not considered as an hypothesis, but the expression of a fact existing in nature.

3. The delirium may be combined with other diseases and injuries, situated in many different parts of the body.

4. When violent, it obscures and renders imperceptible most of the symptoms of the co-existing disease.

5. It is doubtless necessarily accompanied, as all vital excitements are, with an unusual amount of the circulation of the blood in the organ affected; and is, from this cause, sensibly influenced by cups, blisters, and emetics. It is not so far checked by the use of emetics as to render these advisable as a leading means of cure. It is not sufficiently under the control of the general circulation to be cured by venesection, or to be sensibly relieved by it without such an exhaustion as is highly dangerous to life.

6. It is entirely and absolutely under the control of opium; although the fevers and other diseases which are liable to accompany it may be by no means so.

7. It admits of very large doses of opium, which are not productive, either at the time or subsequently, of any injurious consequences, provided they are not repeated after a tendency to sleep is evinced.

8. The patient must *sleep or die.* There is no alternative. Yet the physician should personally watch the effect of very large doses of opium.

9. There is no distinction of stages which need occasion a moment's delay in resorting to opium.

10. Purgatives are of no use in this delirium; but it is necessary to prevent costiveness subsequently to the administration of opium. Purgatives may be necessary for diseases which exist at the same time; but, when this is the case, they are, in general, most advantageously postponed till after sleep has been obtained.

11. Gentle stimulants are frequently useful during the convalescence; but these should not resemble ardent spirits; and an excellent and sufficient one is capsicum. Nor should any ardent spirits, unless indicated by peculiar circumstances, be given during the paroxysm.

---

### MEDICAL JURISPRUDENCE.

Rolfe *versus* Stanley.—The following remarks on the above trial are taken from the *Medical Gazette,* and we entirely concur in the view there given of the subject.

The particulars are as follows: Mr. Rolfe, the son of a tailor in Dean-street, Soho, fell from his horse, and bruised his knee. A general practitioner, who lived in the neighbourhood where the accident happened, having washed and dressed the part, conveyed the patient home, but feeling some doubt and anxiety about the nature and result of the injury, he consulted Mr. Stanley, one of the surgeons of St. Bartholomew's Hospital, and joint lecturer with Mr. Abernethy on anatomy and surgery, a gentleman well known throughout our profession for that combination of zeal, talent, and information, so necessary for a teacher in so eminent a medical school. When Mr. Stanley examined the knee, some hours had elapsed since the accident: the part was much swelled; there were two wounds below the knee; and about an inch and a half from the nearest, on one side of the knee-pan, a hard body, about the size of a nutmeg, could be felt deep under the skin. It must have been one of two things, either a foreign body from without which had penetrated through the wounds, or a piece of bone which had been chipped off from the knee-pan, or from the adjoining condyle of the femur.

The latter appeared to Mr. Stanley to be most probable, and under this idea his treatment was correct. But it was a piece of flint; and, supposing this to have been ascertained at the time, what was to be done? The foreign body had penetrated so far, as to render it probable that it had pierced the capsule, and entered the joint. Now, to have followed it here with a view to its extraction, would have led to such interference with the parts, as must have been attended with a great aggravation of the danger. So that, in either case, the treatment adopted was the only one admissible under the circumstances; because wounds of the knee-joint are always extremely hazardous; a hazard which is much increased by all unnecessary disturbance of the parts.

But, supposing Mr. Stanley had known that it was an extraneous body, it was not near the surface, but had every appearance of being deeply seated, probably under the fascia, and a force capable of driving it in an inch and a half beyond the wound, would likewise have been capable of driving it deep. It was like a bullet in a gunshot wound; and it is settled practice in such wounds, that it is bad surgery with probe and knife to grope after the bullet. "The extraction of foreign bodies," says Mr. Samuel Cooper, "ranks as one of the most urgent motives for the dilatation of the wound, and no doubt it is right to remove at first as many of them as possible. Their lodgment irritates the wound, causes violent nervous and inflammatory symptoms, and copious suppuration; circumstances which the timely extraction of them may prevent. Yet let it be remembered that the extraction of foreign bodies is frequently attended with immense irritation, and that, while they lie too firmly fixed in parts, it is often a matter of impossibility. After the sloughs have separated, and the wound has become widened, suppuration frequently does not prevail long before the extraneous substances become loose, spontaneously approach the skin, and easily admit of removal without any dilatation. Hence it is generally prudent to extract at first only such foreign bodies as are near the external opening, quite loose, and removable without much irritation; or such as press on parts of importance, and thereby excite dangerous symptoms. The surgeon should avoid interfering with those which are deeply and firmly lodged in the wound. He should await suppuration and the detachment of sloughs, and, when the foreign bodies become moveable and apparent, he should extract them with or without an incision, as circumstances may demand. The examination of the wound ought to be made as much as possible with the finger, which irritates less, and feels more distinctly than a probe."*

Mr. Abernethy, speaking on the same subject, says, "It is very impertinent and injurious to be searching after bullets: the common prejudice is all on the other side of the question; the patient will say, 'What! have you not extracted the bullet yet? Oh, how much I wish it out.' Indeed, we meet with it in almost every novel, that Sir Harry Somebody has fought a duel, and has been shot, but the bullet has been extracted, and hopes are entertained of his recovery; as if the hopes of his recovery all depended upon the extraction of the ball. Now, I have been in the habit of mentioning a case here, to show the inutility of searching for bullets. Sir Ralph Abercrombie received a musket-shot on the upper part of the thigh-bone; the ball penetrated through the muscles, fascia, and so on, and lodged in the upper part of the trochanter major; the surgeons were very anxious to extract the ball, and made many attempts to do so, enlarged the wound, and probed and searched for the ball: however, they did not extract it, and the General, on his passage home, died." [The wounds made in searching after the ball became affected with erysipelatous inflammation, and thus occasioned death.] "The surgeon of the ship, after he was dead, examined the wound, and found the ball sticking so firmly in the trochanter major, that he was absolutely obliged to remove a portion of the bone with a trephine before he could get it out."

Even, therefore, if it had been known that the foreign body was a stone, it would have been hazardous practice to have probed for it and cut it out. The result of the case is surely in favor of the gentler treatment. The tailor is alive and well, and can bend his knee. The result of the opposite practice might have been an opening into the joint, inflammation and destruction of it, loss of limb or loss of life.

Mr. Stanley pursued the former practice. A splint was applied to prevent the motion of the joint, and the best remedies were employed for subduing inflammation. The inflammation subsided, leaving the knee, however, somewhat swelled, and the hard body still to be felt under the skin. At this period the patient went for the recovery of his health to Hammersmith: an imprudent use of his limb (he went to the Duke of York's funeral,) occasioned a return of inflammation; and Mr. Lilly, a general practitioner, was consulted ten months after the accident. This gentleman, when he first examined the knee, had no suspicion of what the hard body really was; he, like Mr. Stanley, supposed it to be a broken portion of bone; and it was not till he

* Surg. Dict. p. 549.

had attended the patient two months, and then only because the skin broke, that he discovered its real nature. Hereupon he enlarged the new opening in the skin, and extracted a flint about the size of a nutmeg; two smaller fragments were also removed; the opening soon healed, the swelling of the knee subsided, the patient recovered the possession of his health and the use of his limb, so that he can now sit cross-legged on his father's board. But, thinking that Mr. Stanley ought to have detected the nature of the hard body and removed it, and that, if this had been done, he should have been saved several months of suffering and disability, he commences a suit against him for unskilful treatment, and the cause was tried on Wednesday, the 13th of February. Mr. Cross was employed for the plaintiff, Mr. Sergeant Taddy for Mr. Stanley, and Judge Burrough presided. Sir Astley Cooper, Mr. Abernethy, Mr. Bell, Mr. Brodie, Mr. Travers, and Mr. Green, were subpœnaed by Mr. Stanley, and testified the high opinion they entertained of his ability and knowledge in his profession; and that, if they had been consulted about the case, they should have formed the same opinion, and employed the same treatment. The only medical men examined on the side of the plaintiff were the two general practitioners; the one who was called to him on the first occurrence of the accident, and who stated that Mr. Stanley examined the knee with the utmost degree of attention; and the other who extracted the flint: and this gentleman acknowledged that, when he first saw the knee, he concluded that the hard body was a piece of bone, and that he did not suspect what it was till two months afterwards, when the skin gave way and uncovered it.

Thus, although an error was certainly committed in the original opinion of Mr. Stanley, yet there was uniform and overwhelming evidence that this error was not the result of that neglect or that ignorance which the law of the land considers an object of punishment, but such as any man, even the most eminent surgeon, might, and in fact does, occasionally commit. There was evidence, moreover, that this error did not in the slightest degree alter the result of the case; for had Mr. Stanley known from the beginning that the hard body was a piece of flint, he would not have interfered with it. Judge Burrough summed up to this effect, and almost every body in court so fully anticipated that the plaintiff would be nonsuited, that the reporters of the evening papers, who left the court before the verdict was returned, ventured to publish it as given in favor of the defendant; but, to the astonishment of every one, the jury retired, and, after being absent an hour, returned a verdict of £30 damages against Mr. Stanley.

An eminent counsellor being consulted about a claim, wrote as follows: "I am clearly of opinion that there is no claim either in equity or law, but, as it is impossible to tell how the evidence may impress a jury, I advise it to be tried." And an eminent equity judge once said in our hearing, "I think I had better decide, rather than send the question to the toss-up of a jury." So much for the boasted trial by jury, far more valuable as a means of protecting the liberty of the subject against the oppressions of government, than a means of insuring justice between man and man.

On the unexpected and extraordinary result of this trial, we shall say nothing to the barristers* and the judge, because the former confined themselves

* Mr. Cross talked a great deal of nonsense about the splints being put on to compress the supposed piece of bone against that from which it had been broken; whereas they were applied, as they always are, to prevent the motion of the joint.

to their duty, and the latter performed his; but what shall we say to the plaintiff, the jury, the law, and the public? We will grant, what there is no doubt about, that Mr. Stanley's opinion with regard to the nature of the foreign body turned out to be erroneous; and we will grant also, although there is absolute proof to the contrary, that the error of opinion occasioned an error of practice, and thus led to the suffering and disability of the patient; and then we will ask, whether it is just that the medical should be the only one of the learned professions in which an error of judgment is punishable by law? A statesman may commit blunders in the management of the currency, and by so doing raise such a hurricane in the commercial world as strips thousands of the most opulent bare to the very branches, and reduces them for life to poverty and wretchedness. A clergyman, from preferring dining out and fox-hunting to his parochial duties, or even a well-meaning priest, for want of a "searching and awakening" power in his discourses, may so neglect or misconduct his spiritual duties that his parish abounds in immorality and thoughtlessness, and shoals of souls are lost for want of proper and penetrating instruction in religion. A barrister, from ignorance of his brief, or want of zeal in his cause, from ignorance of law, or from defective powers of statement, may lose a cause, which a more zealous, learned, or eloquent counsel might have gained. In all these cases, the error is punished only by the censure of those who suffer from it, or whose minds happen to be directed to it; but if a tailor tumbles from his horse and gets a flint into his knee, and the surgeon mistakes the flint for a piece of bone, he is prosecuted for the error of opinion, though it leads to no error of practice, and twelve men, who know no more on the subject than they do about the philosopher's stone, condemn him to a heavy fine. Do the public suppose that medicine is so much easier than legislation, theology, and law, that physicians and surgeons ought to be always right, or, if occasionally wrong, they can be so only from carelessness or ignorance so gross as to deserve legal punishment? On the contrary, the public ought to know that it is so difficult that it has been emphatically called "a conjectural art," and that no human being, whatever may be his attention, knowledge, and judgment, but must sometimes err, and the error may involve health, and even life. It not only is so now, but it must continue so, as long as man is man, and human nature is liable to erroneous opinions. For many years we have had frequent intercourse with the most eminent physicians and surgeons of our time, some dead, others still alive, and have observed them engaged in their practical duties, in the sick room by the bedside, over one or other of those bodily afflictions which it is our painful lot to live among, and which we exert our best efforts to relieve; and we do not remember one among them, however high his eminence, or great his knowledge, who has not been sometimes wrong, and thus occasioned more or less injury to the patient. Our profession brings enough of toil and of anxiety, without the additional evil of being liable to legal prosecutions. Of all the learned professions, or, to speak more generally, of all the pursuits in which learning or science is applied to practical purposes, the medical profession is the most oppressed by the public and the law. We are persecuted in the prosecution of our anatomical studies; imprisoned or fined for procuring dead bodies by stealth, although we have no other mode of obtaining them; and then, if we commit an error in the practice of our profession for want of this knowledge, we are held up in a court of justice to the gaze of the public, clawed by some tiger of a barrister, and heavily fined. Thus, we are punished for the pursuit of knowledge, and then punished for the want of it. In eastern countries, if the patient dies they cut off the doctor's head. In England, they leave his head upon his shoulders, but they cut up his reputation, which is almost as bad.

---

IMPUTATION OF POISONING.—*Detection of a Conspiracy, by the inconsistence of the evidence for the prosecution with the chemical analysis and with the effects of poison on the system.*

CASE I.—Samuel Whalley was indicted at the York spring assizes, in 1821, for maliciously administering poison to Martha King, a dress maker, with whom he cohabited, and who was pregnant by him. The indictment contained three charges:—a charge of administering poison for the purpose of killing the woman; another of administering it for the purpose of destroying the living child in her womb; and a third of administering it for the purpose of procuring abortion, the child not being quick.

The moral evidence against the prisoner was derived almost entirely from the testimony of the prosecutrix King, and that of another woman Merryweather, who lodged in the same house with her. Its purport was, that the prisoner had, under promise of marriage, cohabited with King, who became with child in consequence; that the marriage was put off, after proclamation of banns, on the ground of its being inconvenient at the time to the prisoner;—that, on two occasions after this, and recently before she was poisoned, he tried to prevail on her to take drugs for the purpose of destroying the child, and maltreated her because she refused;—that she had a dream about purchasing poisoned tarts, and that a fortnight afterwards the prisoner brought her several tarts, which, while he declined partaking of them himself, he urged her to eat, "because he should like young Samuel (the fœtus in utero) to have one;"—that she did not take any that day, but ate some the following afternoon;—that after she had eaten them, and was taken ill, the prisoner paid her a visit, and in quitting her made a remark which implied he thought her dying, and knew she had taken something deleterious.

The prisoner, on the other hand, while he admitted the cohabiting, the promise of marriage, and even that a day had been fixed for it, stated that it was broken off in consequence of his having caught her in a suspicious situation with another man;—that she frequently threatened his life on account of his refusing to make good his promise to marry her;—that he carried the tarts to her, because she asked for them on account of a curious dream she had about tarts;—and he proved, by two witnesses, that she had arsenic in her possession, although in her evidence she denied she ever had any, or knew what it was. The defence was therefore grounded on a charge of conspiracy on the part of King and Merryweather.

The medical evidence was to the following effect:

According to the deposition of King, corroborated in many points by that of Merryweather, she felt an "unpleasant coppery taste" while eating the first tart, and this was so much increased while she was eating a second that she did not take the whole of it. Half an hour afterwards she became sick, felt a burning heat and pain in the stomach, was seized with trembling and feebleness of the limbs, and subsequently had some vomiting. Her fears being in consequence excited, she sent for Mrs. Merryweather, and along with her examined two tarts which remained; and they found a white powder hidden under the preserves. They both became alarmed, and Merryweather immediately set off in quest of a medical man. But it appeared that, notwithstanding her state of alarm, the messenger went to a gentleman who lived a mile off, who had never attended King before, and to whose house she did not know the way. Mr. Thackrah, surgeon at Leeds, who was the gentleman called, arrived at Mrs. King's house about six in the evening, two hours nearly after she had taken the tarts. He found her agitated, and complaining of the symptoms mentioned above; but "the pulse was not much accelerated, the tongue exhibited nothing particular; she did not complain of urgent thirst, or sense of heat in the mouth or throat; nor did she seem to have acute pain." As she told him she had not vomited, he immediately administered an emetic, and soon afterwards another, as the first did not operate. She then vomited. The matter discharged was preserved in a wash-hand basin, and taken home that evening by one of his pupils who had accompanied him; but Mr. Thackrah admitted that the prosecutrix might have introduced a small quantity of poison into the basin before she vomited.

He left her about seven, and sent his assistant to see her at ten. Meanwhile she had, according to her own account, another attack of vomiting, the matter of which she preserved, and showed to Mr. Thackrah at his visit next day; but she did not speak of it that evening to the assistant. This stuff was not taken away for some days, as Mr. Thackrah did not happen to have any convenient vessel with him at his second visit. On this occasion he found her complaining of headache and pain in the bowels, and the tongue was furred; but she had no particular illness that was apparent, nor was there subsequently any of those effects which are usually caused by small doses of arsenic. After a few days' attendance, deeming her well, he discontinued his visits. She, as well as Merryweather, however, stated at the trial that she had been obliged to keep her bed several days, and that she had never been well afterwards.

The chemical analysis of the various suspected articles was conducted partly by Mr. Thackrah, partly by Mr. Walker, a chemist of Leeds.

In the two tarts, which were delivered to Mr. Thackrah, at his request, on his first visit, they found under the preserves a white powder, which proved to be arsenic. Mr. Thackrah remarked that, when a portion of the powder, with a little of the preserve adhering to it, was dissolved in distilled water, and alkalised with subcarbonate of potass, the sulphate of copper caused a green, and the nitrate of silver a yellow, precipitate; and Mr. Walker determined the quantity in each tart to be between four and five grains.

Mr. Walker likewise examined the matters of vomiting, and detected arsenic by the same tests in both. The quantity in the first was exceedingly minute,—not above one grain dissolved in at least three pints of fluid. The second matters of vomiting contained a much larger quantity,—namely, between twelve and fifteen grains; and, from the observations Mr. Walker made, "he doubted very much whether the arsenic of this analysis had ever been in a human stomach."*

* Mr. Walker having died since the trial, I have not been able to procure all the particulars of his experiments. The fact that arsenic has not been in the stomach may be ascertained by finding that the precipitates procured from the alleged matters of vomiting by the liquid tests, and more particularly by sulphuretted hydrogen, do not yield empyreuma, or exhale the odour of burning animal matter in the course of reduction.

Such were the facts of this important case. The following are the conclusions which may be drawn from the medical evidence:—

In the first instance, Mr. Thackrah deposed on the trial that, although the illness of the prosecutrix was undoubtedly caused by something which had disordered her stomach, he did not think her situation corresponded with that of a person who had taken so much arsenic as was to be inferred from what was contained in the tarts analysed;—and that, if he had heard nothing about poison, he would not have supposed she had taken any. He admitted at the same time that he could not affirm positively, from the symptoms, that she had not taken a *minute* quantity of arsenic, as the symptoms of arsenical poisoning are subject to great variety.

In the second place, Mr. Walker deposed, in regard to the coppery taste said to have been felt by the prosecutrix while eating the tarts, that arsenic could not cause such a taste, because its taste "*is sweet*, not like copper; if mixed with sweet tart, it could not be perceived at all."

Thirdly, according to the analysis of the powder in the tarts which were not eaten, the prosecutrix could not have taken above *ten* grains of arsenic in what she did eat. Now, even after repeated attacks of vomiting, the last matters discharged contained *at least twelve grains;* which clearly showed that arsenic had been mixed with the stuff after it was vomited,—if, indeed, there was vomiting at all. The same inference is deducible with still greater certainty from the comparison of the quantity contained in the last matters of vomiting with that contained in what was discharged a short time before when vomiting was encouraged by emetics and the free use of diluents; the latter quantity not being above *one* grain. The prosecutrix indeed stated that the matter from which the larger quantity was procured had been discharged into the same bason as that from which the former matters of vomiting had been taken, and that a considerable white sediment had been left in the bason when Mr. Thackrah transferred the contents into another vessel. But Mr. Thackrah was sure that no perceptible sediment was left.

Fourthly, Mr. Thackrah deposed, that if so great a quantity had been taken as to render it possible for twelve or fifteen grains to be vomited two or three hours afterwards, it would have occasioned the most violent symptoms, and probably death.

It is impossible to conceive more pointed evidence. The medical investigations were alone amply sufficient to decide the nature of the case.

The prisoner, as will be readily anticipated, was found Not guilty. It may be satisfactory to add, that the prosecutrix King and the witness Merryweather were immediately committed on a charge of conspiracy. But the judge, considering the unfortunate state into which one of them had been brought by Whalley's means, and that they had signed a paper to the effect that all the charges made against him were false, and the result of a conspiracy against him, acceded to Whalley's request to allow them to be discharged.

Poisoning with Arsenic.—*Perforation of the Stomach. Detection of the Poison in very minute quantity. Inefficiency of the liquid tests.*

Case II.—Margaret Wishart was tried at last Perth spring circuit for the murder of her sister Jean and her natural child, by administering to them arsenic or some other poisonous substance.

The deceased Jean Wishart was totally blind, and in consequence had been for some time a burden on her sister, the prisoner, who in particular always made ready her food. The blind woman had a lover in the person of a man, Roy, a lodger in the house, by whom she had a child about four years before her death, and by whom she was again pregnant at the time the poison was supposed to have been given. This man was also on very intimate terms with the prisoner, and there was strong reason to believe that he cohabited with her. Such being the state of the household, it may be conceived that the several members of it could not be on good terms with one another. Accordingly, although several neighbours bore testimony to the friendly disposition of the prisoner towards her sister, those who lived in the house with them deposed that the deceased frequently complained of the usage she received both from her sister and from Roy; and she asked one witness to take her as a lodger, as, on account of her pregnancy, she was apprehensive of ill treatment at their hands, and even used the expression that "she would not get leave to live."

On the evening of Tuesday the 3d of October, she was seen by a neighbour taking porridge to supper; the prisoner and a man who lodged in the house being present, but not joining her. About twenty minutes afterwards she felt unwell, and blamed the porridge as the cause; and in a few minutes more she was attacked with vomiting. During her subsequent illness, which ended fatally on the evening of Saturday the 7th, she had not any medical attendant, except that on Friday, when she was taken in labour, and a midwife sent for to deliver her. Several people advised the prisoner to get a medical person to attend her, and the deceased herself asked her to do so; but she refused, saying that she could not afford it, and that all the doctors in Arbroath could not do any good. She likewise refused to send for another sister who lived in the same town.

A few weeks before her trial, the prisoner added an important circumstance to the moral proof, which, it will be seen, was previously by no means strong, by attempting to suborn a witness. A young woman, who visited her in prison, said, in presence of the jailor and others, that she had accompanied the deceased to various shops in Arbroath, to ask for poison, and that the deceased actually got arsenic at one of them; but on the trial she confessed that it was all a fabrication, and that she had been prevailed on to tell it by the prisoner's repeated importunities.

No evidence could be procured that the prisoner had either purchased arsenic or had it in her possession.

The medical evidence in this case rested chiefly on the morbid appearances and chemical analysis.

The account which could be procured of the symptoms was exceedingly imperfect, as it merely went to show that the deceased was affected with more or less vomiting, purging, thirst, and general uneasiness, from the evening on which she was taken ill till that on which she died; that she was delivered of a living child on Friday; and that on Saturday morning the limbs were cold and stiff. An important point regarding the symptoms, however, was ascertained, namely, that they began within half an hour after a meal. The symptoms do not appear to have been violent. Such as they were, the witnesses for the crown all agreed that they might be caused by arsenic, but admitted that they might equally be caused by natural disease.

The body was disinterred for examination on the 15th, eight days after death, by Drs. Arrot, Sharpey, and Palmer; and the following is an account of the chief appearances described in their report: On opening the abdomen, they found about a pint of red-coloured liquid in the cavity of the peritoneum; a small perforation on the anterior surface of the stomach, through which a small quantity of black fluid escaped; the external surface of the intestines red and highly vascular; the uterus not reduced to its natural size; the other viscera healthy. The stomach contained a thick, opaque, brownish black liquid, and a little solid vegetable matter. Its inner surface was very vascular, and marked in different places with dark brown spots of various sizes; and the villous membrane was abraded here and there. The intestines were internally very red, and quite empty.

A portion of the stomach, including the perforation, together with part of the contents, was sent to me for examination and analysis. The stomach having been preserved in a bottle along with the contents, and the examination not having been made till the 21st, the vascularity had entirely disappeared, and the membranes had imbibed the fluid like a sponge; but the nature of the perforation was quite distinct. The aperture in the peritoneal coat, which was about the size of a pea, was surrounded by a dark ragged margin, and the inner coats were more extensively destroyed around it.

The chemical evidence was decisive of the presence of arsenic in the fluid part of the contents, and still more in the coats of the stomach.

Drs. Arrot, Sharpey, and Palmer, made several solutions by diluting and filtering the contents, and by boiling portions of the stomach and filtering the decoctions. In none of these fluids did the ammoniacal sulphate of copper, or ammoniacal nitrate of silver, indicate the presence of arsenic. But the sulphureous test, in all its forms, gave satisfactory indications. After some preliminary trials with sulphuretted hydrogen water, and the hydrosulphuret of ammonia with the subsequent addition of muriatic acid, they united the various fluids, and subjected them to the process which I have recommended.* The liquor was acidulated with acetic acid, and then subjected to a stream of sulphuretted hydrogen; upon which a yellow precipitate was procured, that yielded by reduction with the black flux, in three different trials, patches of metallic incrustation. I regret that the limits of this abstract do not permit me to give more than the substance of the able report of the Arbroath gentlemen. The whole investigation reflected the greatest credit on them, but particularly the chemical part of it; for, if I may judge from my own experiments, there has never been a judicial inquiry in which so minute a quantity of arsenic has been so unequivocally discovered.

In my own analysis, I first formed a decoction of the contents and of the stomach separately, and subjected each, after acidulation with acetic acid, to sulphuretted hydrogen gas: a lemon-yellow milkiness ensued, which, on boiling, gave place to a lemon-yellow flocculent precipitate, with brilliant brass-yellow scales intermingled. This, when collected from both fluids, and dried and reduced in a small tube, gave an arsenical crust with all its properties fully developed; and the crust was converted, by repeated sublimation, into little octahedral crystals of oxide of arsenic, which I estimated as amounting to about a fortieth part of a grain.

As the decoction of the stomach still retained its yellow colour, I evaporated it, and procured a yellow precipitate in broad flakes. Knowing from expe-

* Edinb. Med. and Surg. Journal, for July 1824; and Edinb. Medico-Chir. Trans. vol. i.

rience that there was too much animal matter present in this precipitate to admit of the process of reduction being applied directly, I subjected it to a farther process, in order to destroy the animal matter. The impure sulphuret was acted on by nitric acid with the aid of heat, and consequently oxidated; the acids were then neutralised with potass, and the solution evaporated to dryness; the mass was then deflagrated in a glass tube, which process takes place gently, with effervescence and without burning, if the proportion of the nitre is not too small; the product was then dissolved out, exactly neutralised with nitric acid, filtered, a small portion tested with nitrate of silver, which gave a brick-red precipitate, and the remainder treated with a solution of nitrate of lead as long as any precipitation took place. The precipitate, which, besides other salts, contained arseniate of lead, gave a remarkably characteristic crust when mixed with freshly ignited charcoal and heated in a tube strongly with the blowpipe; and the crust subsequently gave octaëdral crystals when repeatedly sublimed.* The quantity of oxide procured by this process was not sensibly greater than by the former.

The prisoner was convicted of poisoning her sister. Many persons entertained doubts of the sufficiency of the moral evidence, and some attempts were made to procure her pardon on the ground of its inadequacy. She was executed, however, and died protesting her innocence.—(See a paper by Dr. Christison, in the *Edinburgh Med. and Surg. Journal.*)

---

*Spots of Blood.*—M. Adelon lately read an Essay of M. Raspail to the Academy of Medicine in Paris, upon the chemical and microscopic phenomena, by which the presence of blood upon linen cloth and instruments of iron may be detected. The author assures us that he has met with all the characters given by MM. Lassaigne and Orfila, especially the last, by submitting linen rags dipped, not in blood, but in a mixture of white of egg and infusion of madder, to the tests they have indicated. He considers it probable that the spots made upon linen by the greater number of red fruits, in which albumen is found mixed with a colouring principle, would afford the same results: he thence concludes that the chemical proofs at present given as the tests of the presence of blood, or of its colouring principle, have so little of certainty in them, that the medical man cannot presume to give any legal evidence concerning them. With regard to the microscopic characters, M. Raspail informs us, after numerous observations, 1st, that the globules vary in diameter according to the organs which supply the blood under examination, contrary to the general opinion, which considers their diameter as constant and invariable in every part of all individuals of the same species. 2dly. These globules are colourless, and ought not to be considered as composed of an exterior coloured covering, and of a central white nucleus. It is sufficient, in order to satisfy oneself of the fallacy of this opinion, to dilute the drop of blood to be examined in a greater quantity of water; the colouring matter is then seen to spread itself upon the edges of the glass, and the globules remain of a white colour, undiminished in size. 3dly. These globules

* I have since been in the custom of employing a different process for getting rid of the animal matter *before* the precipitation of the sulphuret; and in consequence the sulphuret, under all circumstances, is procured in a state fit for immediate reduction. This improvement I shall soon have an opportunity of making public.

are, in fact, only albumen in a minute state of division: they are generally soluble in ammonia; are precipitated by acids; and are dissolved in small quantities after a considerable period in water.

Such are facts attending the examination of recent blood: as to that which is dried upon linen cloth, and afterwards soaked in water, the microscope shows nothing positively. In fine, microscopic observation does not offer, any more than chemical analysis, any certain means of ascertaining the presence of blood.

M. Orfila's answer to the above remarks is as follows: "My opinion corresponds with that of M. Raspail as to the microscopic characters, and I have said so in my essay. As to the chemical tests, I have long been acquainted with the experiments of M. Raspail; I have repeated them all; I declare that they are all wanting in exactness ; and I request a commission to be appointed, of which I shall not be a member, which shall be directed to repeat these experiments, as well as those made by me, to decide between us."—The commission is appointed, and consists of MM. Mau, Adelon, Delens, and two other members whose names are not mentioned.

### PHARMACY.

*Ergot of Rye.*—In a Memoir on the Employment of Ergot of Rye, lately published by M. Villeneumay, he considers the best mode of giving the medicine to be in powder, in a dose of about twenty grains, and repeated at the end of half an hour or an hour, till its effects become apparent. He thinks wine the best vehicle for administering it, and, when wine disagrees, he recommends its being mixed with syrup of peppermint. The decoction of this substance has also been given as an enema, and with great effect. The power that this medicine has over the contractions of the uterus seems to point it out as useful when hemorrhagy succeeds delivery, and where a contraction of the uterus would put a stop to it. In such cases it has been tried, and with great advantage. *(Revue Med.)*

### MIDWIFERY.

*Laceration of the Uterus.*—Mr. Birch makes the following interesting remarks, in the *Medico-Chirurgical Transactions.*

Laceration of the uterus proves fatal in one of three ways: either the woman dies very speedily from the disturbance of the vital functions immediately following the injury, and which no doubt is brought about by the intimate sympathy between the uterus and the brain; or death happens less speedily, but at an uncertain period, from the consequent inflammation of the uterus, peritoneum, and abdominal viscera; or, surmounting all these dangers, the woman eventually sinks from an incapacity of her natural powers to repair the injury sustained.

With all these difficulties and dangers in view, we could not have felt surprised if recovery had never taken place. Women, however, have sometimes survived when the laceration has been of the cervix of the uterus, of the vagina, or of both ; but never, so far as I know, when the body or fundus of the womb has been implicated.

From comparing the result with the injury sustained in the two cases I have reported, a question arose in my mind, whether any satisfactory reason could be given why laceration of the anterior part of the cervix uteri and vagina should prove less fatal than laceration of their posterior part? That such is the fact, I satisfied my mind, by looking to all the records of successful cases I have been able to discover, and finding that in all the laceration was either to one side or to the front.

It appears to me that the intestines or bladder must of necessity come into contact with the wound when at the fore part, and do not only serve to prevent a further effusion of air or blood into the abdominal cavity, but perform a much more beneficial part, by throwing out coagulable lymph, receiving this natural glue poured out by the edges of the wound, in this way becoming adherent to them, and forming their temporary bond of union, until granulations have sprung up, contracted, and cicatrised, the whole of which process also this arrangement puts in the most favorable train. Whilst at the back, the rectum being too closely bound down to the sacrum, there is neither bladder nor intestine to close the wound, and hence its whole extent has to be filled up by granulations heaped upon granulations; and before nature is enabled to accomplish her task she sinks, exhausted by the constant source of irritation, or falls a sacrifice to an attack of inflammation consequent upon it. Whether this explanation will prove as satisfactory to the minds of others as it has done to my own, I am of course unable to divine: the fact however is, that lacerations of the posterior part of the vagina and cervix uteri have always proved fatal.

Recovery from laceration of the front or sides of the cervix uteri and vagina, has taken place under very different circumstances. It has happened when the child has not passed into the abdomen, but has been extracted from the uterus; and it has happened when the child has passed into the abdominal cavity,—when it has been allowed to remain there,—when it has been extracted through an opening made in the abdominal parietes,—and when it has been brought back through the laceration, and delivered "per vias naturales."

When the child has passed into the cavity of the abdomen, and has been allowed to remain there, it is not to be denied that women have lived, but their recovery has almost invariably been so imperfect, that they have for years dragged on an existence miserable to themselves and burthensome to their friends,—the children decaying, and being discharged by abscess slowly and painfully into the intestines, or, together with fæces, through the abdominal parietes; or their recovery has only been for a short time, at the end of which they have been attacked by inflammation, and died. Whilst, when the women have lived after delivery by turning or gastrotomy, the recovery has been perfect: so perfect, that they have in several cases subsequently borne children in safety.

The cases of recovery under any circumstances have been so rare, and so few of the unsuccessful ones have been recorded, that we cannot positively state gastrotomy has saved a greater proportion of lives than delivery by the natural passages; or that this latter method has been more successful than gastrotomy: but that one or the other method is to be preferred, according as it is practicable with less mechanical injury to the woman than the other, is a position to which all must assent.

From the foregoing facts I am of opinion the following principles are deducible:—

1. That delivery ought always to be accomplished, inasmuch as it certainly does not lessen, but I believe increases, the chance of the woman's surviving, if she lives it insures her a more perfect recovery; and moreover it affords the only, although confessedly remote, prospect of saving the child's life.

2. If the child remain in the uterus, it ought to be extracted by the forceps or lever, if possible; by the perforator, if these will not succeed; or by turning, if neither kind of instrument is applicable. The forceps are to be preferred, for a very obvious reason; and embryotomy is preferable to turning, because the pelvis being almost always small, this latter operation would often not succeed, and it would endanger or insure an increase of the mischief.

3. If the child have but partially escaped into the cavity of the abdomen, it ought to be brought back, if we can accomplish it without the greatest violence; and if this be not possible, it must then be taken through an opening in the abdominal parietes.

4. If the child have entirely escaped into the cavity of the abdomen, it should be brought back through the laceration, if practicable, without great force; which I believe will always be the case when the laceration is of the cervix or vagina, and when the operation is undertaken within any reasonable time from the period of the accident.

5. That where turning is not practicable, from the firm contraction of the uterus, or where it would endanger a great increase of the rent, there gastrotomy should be had recourse to, as affording the woman a better chance of living, a much better chance of perfect recovery, than a total abandonment of her to the "vis medicatrix naturæ," and as giving her child the sole chance of surviving.

6. That whatever is done should be done speedily, for these reasons: if the child have not already passed into the cavity of the abdomen, the probability is that it will very soon; if the child have passed into the cavity of the abdomen, the shorter time it is in contact with the viscera the less likelihood will there be of subsequent inflammation, and the less chance will there be of the uterus contracting firmly.

---

### CHEMISTRY.

*On a New Acid existing in Iceland Moss.*—The reddish purple colour which is produced by adding a decoction of Iceland moss to per-salts of iron, has been attributed to the presence of gallic acid, but is found by M. Pfaff to be occasioned by a new acid body, which may be separated in the following manner:—A pound of the lichen, cut small, is to be macerated in solution of carbonate of potassa, until all that is soluble is separated: the above quantity will neutralise two drachms (about 120 grains) of the carbonate. The filtered liquor is to be precipitated by acetate of lead, and the brown precipitate produced, when well washed, is to be diffused through water, and sulphuretted hydrogen passed through it until all the lead is separated. The filtered liquor is acid, and, by spontaneous evaporation, yields dendritic crystals. The crystals, when heated, carbonise, but produce no odour like that of tartaric acid, and lime is left. If they be dissolved and acted upon by alkaline carbonates, carbonate of lime is thrown down, and alkaline salts, containing the new acid, are produced.

The potash salt crystallises in quadrilateral prisms, needles or plates, and is not deliquescent. The soda salt has similar characters, and the ammonia salt crystallises in needles. These salts abundantly precipitate the acetate and muriate of iron of a red brown colour; they precipitate sulphate and nitrate of zinc white; muriate of manganese slightly of a clear brown colour; barytic and strontian salts abundantly white: being mixed with strong solutions of muriate or acetate of lime, they gradually produce an acicular crystalline white precipitate; acetate of silver yields an abundant white precipitate, which does not change colour in less than twenty-four hours; they do not precipitate salts of glucina, magnesia, alumine, uranium, nickel, copper, cobalt, gold, or platina. This substance has been named the Lichenic Acid, and is distinguished from the boletic acid by the different character of its vapour, and by forming an insoluble salt with baryta.—(*Bull. Univ.*)

---

### MISCELLANEOUS.

*Midwifery in different Countries.*—"According to the account given me by many different persons, unnatural labours are not by any means uncommon, and many women lose their lives in consequence of them; far more instances of the kind occur here than is usual in warm climates. The women are extremely fruitful; it is not unusual to find families of fifteen or twenty children." (LANGSDORFF, vol. i. p. 69.)

The women here always sit in the eastern fashion; but this cannot be the cause, for the greater part of the world do the same, and in countries where parturition is easiest.

"The measles had been very prevalent and very fatal among the Indian women. Almost all the Indian women who caught the measles while they were pregnant, miscarried. Childbirth seems remarkably easy here among the Spanish women. Madame Arguello told me that since she had been in the country, which was a considerable time, she having herself had fifteen children there, she did not recollect having heard an instance of a Spanish woman dying in childbirth. There are no midwives; some female friend assists at the delivery. Many of the Indian women, on the contrary, die at this time, owing, as it should appear, to a very pernicious custom they have of putting some great weight upon the belly during the labour, under the idea of facilitating the delivery. Miscarriages, from the third to the seventh month, are by no means unfrequent among them." (ID. vol. ii. p. 210.)

These Indians, it should be remarked, are a very diminutive race, the men not being above five feet in height: a singular fact, the climate being good, and food in abundance.

"Among the Abissones (an equestrian tribe), the women ride like men, and are said, for that reason, to be subject to long, difficult, and dangerous parturition." (DOBREZHOFFER, vol. ii. p. 120.)

"Among the Spaniards of Paraguay and the Plata.—Comme il y a beaucoup de femmes qui accouchent toutes seules, et que toutes ne savent pas nouer le cordon ombilical, j'ai vu un assez grand nombre d'hommes et de femmes adultes, qui avaient un nombril long de quatre pouces, et qu'on aurait pris pour toute autre chose; ce nombril était mou et enflé." (AZARA, vol. ii. p. 301.)

In 1783, the town of Oppedo, in Naples, was destroyed by an earthquake.

"So remarkable were the effects of this earthquake on the human organs, that in the two following years the women either did not conceive, were prematurely delivered, or brought forth dead children; and of those that were born alive many immediately expired." (Stolberg's *Travels*, 8vo. edition, vol. iii. p. 330.)

"In Scylla, a very opposite effect was produced by this dreadful visitation of nature. Barren women, and those who had left off childbearing, again became fruitful. Some of the former had married, and had remained childless till they were nearer the age of fifty than forty." (*Ibid.* p. 337.)

"I have since been informed that the barren women of Messina, as well as some who had left off childbearing, became fruitful after the earthquake." (*Ibid.* p. 339.)

Stolberg guesses that earthquakes are connected with electrical phenomena; that the excess of electric influence produced the one effect at Oppido, where the shock was strongest, and, in less degree, the directly opposite consequence at the other places.

